# Effect of Suture Length on the Incidence of Incisional Hernia and Surgical Site Infection in Patients Undergoing Midline Laparotomy: A Systematic Review and Meta-Analysis

**DOI:** 10.7759/cureus.34840

**Published:** 2023-02-10

**Authors:** Sulakshana Sekhar, Nishith M Ekka, Rahul Nair, Vinay Pratap, Mrityunjay Mundu, Amit Kumar

**Affiliations:** 1 General Surgery, Rajendra Institute of Medical Sciences, Ranchi, IND; 2 Surgery, Rajendra Institute of Medical Sciences, Ranchi, IND; 3 Internal Medicine, Rajendra Institute of Medical Sciences, Ranchi, IND; 4 Laboratory Medicine, Rajendra Institute of Medical Sciences, Ranchi, IND

**Keywords:** surgical site infection, incisional hernia, incidence, midline laparotomy, ratio, wound length, suture length

## Abstract

The development of an incisional hernia is a common complication of midline laparotomy. Improper fascial closure techniques have a significant role in its development. It can also lead to poor wound healing and increase the risk of developing surgical site infections (SSI). Upon conducting a thorough literature review, various studies have been conducted on closing abdominal wounds. However, there is a dearth of studies portraying the role of suture length in the prevention of incisional hernia and SSI. The effect of using a greater suture-to-wound length ratio on postoperative outcomes was not clearly analyzed or described. The objective of this study is to assess the effectiveness of using a suture length to wound length ratio ≥4:1 versus a ratio <4:1 in preventing postoperative complications such as incisional hernia and SSI.

This study is a systematic review of randomized controlled trials on abdominal wound closure using a suture length to wound length ratio of ≥4:1 and <4:1. published in PubMed, Google Scholar, and Cochrane library. The inclusion and exclusion criteria were defined. The relevant studies identified from 1991 to 2017, were included in the analysis. The primary endpoint was the incidence of incisional hernia, and the secondary outcome was the incidence of SSI.

This meta-analysis considered five randomized controlled trials that compared the effects of using different suture length to wound length ratios during abdominal closure on incisional hernia and SSI. Participants ranged in size from 100 to 363. The trial follow-up period ranged from a minimum of 10 months to five years. The outcomes studied in the two groups were incisional hernia and SSI. The relative risk of the occurrence of incisional hernia if the suture length to wound length ratio was ≥4:1 was 0.42 with a p-value of <0.001 which was considered significant. This implied that using a suture length of more than four times that of the wound i.e., 4:1, significantly decreases the risk of developing an incisional hernia by more than half. The relative risk of developing a SSI was 0.98 with a p-value of 0.966. Thus, this method of abdominal closure uing a longer suture length to wound length ratio does not contribute to an increased incidence or significant change in the risk of developing SSI.

## Introduction and background

Incisional hernia is a major cause of surgical morbidity globally with an incidence between 2% to 20% [1]. Nho et al. observed that incisional hernia was more common after laparotomy (9.9%) than laparoscopy (0.7%) [1]. Among all abdominal incisions, midline incisions had a much greater risk of developing an incisional hernia. The patient and technical factors are important causes in its development. Age, obesity, malnutrition, connective tissue disorders, improper fascial closure, and certain surgeries like bariatric surgery contribute to improper wound healing and leave the patient at a higher risk of developing a hernia [2,3].

Jenkins, in the year 1976, proposed the concept of using a suture length at least four times the length of the wound to prevent fascial disruption [4]. The commandments of safe abdominal closure enumerated by Chintamani demonstrate modalities of prevention of incisional hernia and wound dehiscence by using continuous bites without tension or interlocking that cause a spring coil effect [5].

Incisional hernia and surgical site infection (SSI) prove to be a menace in the postoperative period leading to major morbidity and mortality. Development of incisional hernia requires additional surgical procedures and associated infirmity. Surgical site infection is a common postoperative complication associated with negative economic impact, extended postoperative hospital stay, readmission, sepsis, and even death [6]. To combat this, we need to formulate proper wound closure techniques and abide by them. However, a standard protocol is difficult to design due to the limited research and publications on this subject. From this meta-analysis, we aim to analyze the impact of suture length to wound length ratio on wound healing and its complications. Our objective is to determine if a suture-to-wound length ratio of more than 4:1 can reduce the incidence of incisional hernia without increasing the risk of SSI. Barring the studies included in this review, there is not much data to support the advantage of using one method of abdominal closure over the other. Thus, it is imperative to analyze the different studies to devise a safe method of abdominal closure and a definite protocol that can be recommended to decrease complications like an incisional hernia.

## Review

Aim & objectives

This is a systematic review and meta-analysis of randomized control trials from 1991 to 2017 by applying the population intervention comparison outcome (PICO) structure. The study population includes patients who underwent a midline laparotomy. The intervention analyzed is the abdominal closure with a suture-to-wound length ratio of 4:1 or more. And is compared to an abdominal closure with a suture-to-wound length ratio less than 4:1. The primary outcome was the incidence of incisional hernia, and the secondary outcome was the incidence of SSI following midline laparotomy.

Data source

A systematic search of PubMed, Google Scholar, and Cochrane library was carried out. The search resulted in the inclusion of only randomized control trials between 1991 and 2017 with no language restriction. The relevant studies were reviewed, analyzed, and included in the meta-analysis after being deemed suitable. Medical subject headings (MeSH) search was done using keywords ((midline laparotomy) AND (incisional hernia)) AND (suture length to wound length ratio), and the results were considered. 

Study protocol

An initial search and review of the literature were done. As a result, the protocol was registered in Prospero (CRD42022352205) on August 19, 2022 [7].

Inclusion & exclusion criteria

This meta-analysis intended to include randomized controlled trials, case-control, retrospective studies, and comparative non-randomized studies. However, due to the limited number of studies on this topic and strict inclusion criteria, only a few well-designed randomized controlled trials conformed to the requirements. Studies fulfilling the following inclusion criteria were included: 1) patients having undergone a laparotomy via a midline incision; 2) patients having undergone elective and emergency laparotomy; 3) studies where patients were divided into two groups, one where abdominal closure was done with a suture length more than four times the wound length, and the other where the suture length to wound length ratio was less than 4:1; 4) incisional hernia and/or SSI as outcomes of the studies.

**Figure 1:**
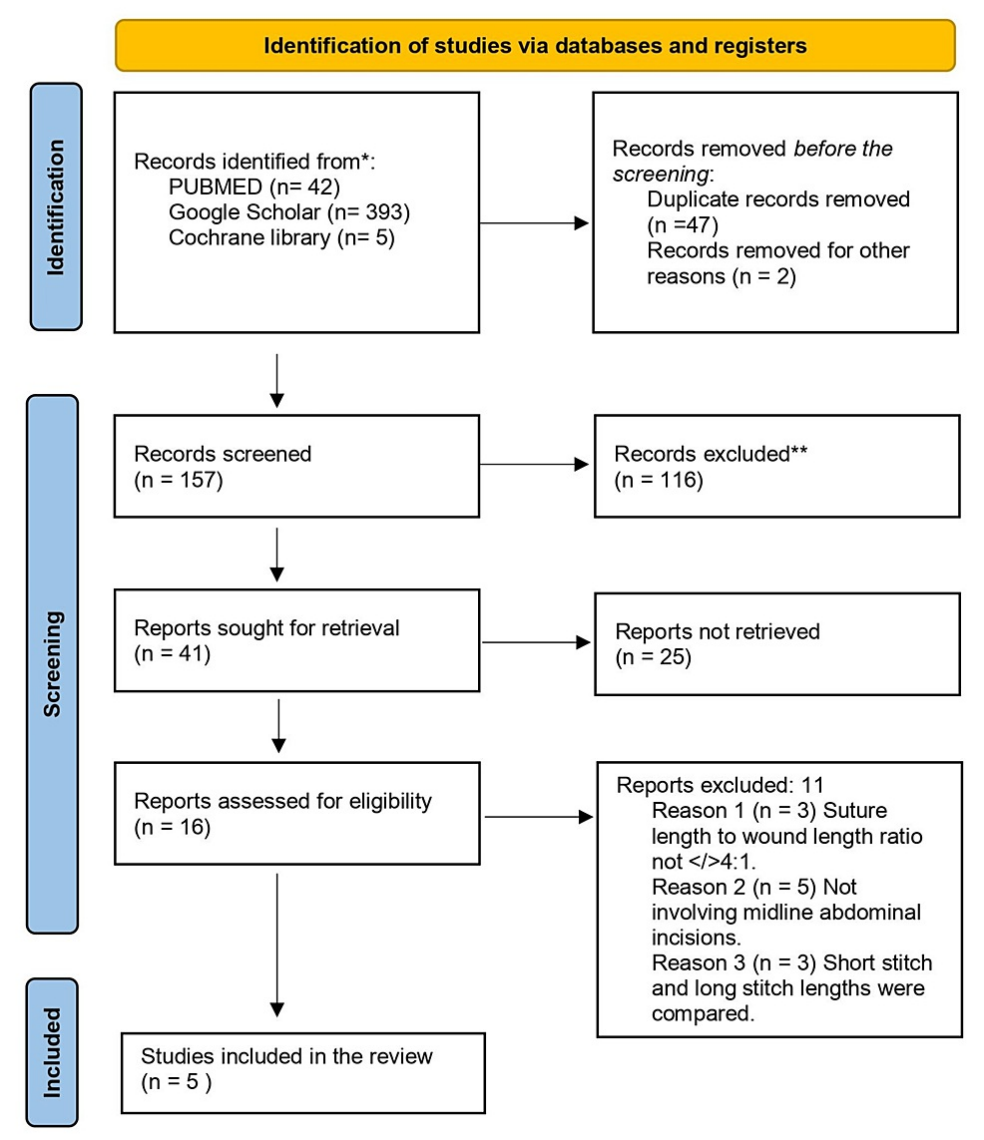
PRISMA 2020 flow diagram showing the selection of the studies analyzed in this meta-analysis PRISMA: Preferred Reporting Items for Systematic Reviews and Meta-Analyses

A total of five randomized controlled trials from 1991 to 2017 were found eligible and assessed for outcomes. The participant characteristics of patients in these studies are those who underwent midline laparotomy and abdominal closure. The procedure of selection of studies was based on the Preferred Reporting Items for Systematic Reviews and Meta-Analyses (PRISMA) and is shown in PRISMA 2020 Flow Diagram (Figure 1) [8].

Data extraction and analysis

Data extracted from the studies included the sample size or number of patients in each group (one group being abdominal closure with a suture length four times the wound length and the other group where the suture length was less than four times the length of the wound), incidence of incisional hernia in each group, incidence of surgical site infection in the groups, and patient characteristics such as age and sex ratio (Table 1).

**Table 1:** Characteristics of the studies included in the meta-analysis

S. No.	Authors	Year	Sample size Group A >or= 4:1 INTERVENTION	Sample size Group B < 4:1 CONTROL	Incidence of incisional hernia Group A INTERVENTION	Incidence of incisional hernia Group B CONTROL	Incidence of SSI in Group A INTERVENTION	Incidence of SSI in Group B CONTROL	Other outcomes	Mean age	Sex ratio (M:F)	Follow-up period
1	lsraelsson et al. [9]	1993	122	241	11	57	8	8	Incidence of contamination, Suture material	58.5	190:173	12 months
2	Williams et al. [10]	2017	76	24	7	6	6	6	Race, BMI	59	42:58	32 months
3	Kendall et al. [11]	1991	104	108	7	7	12	9	Obesity	62.5	84:128	18 months
4	Millbourn et al. [12]	2009	495	27	56	7			Operating time, BMI	64.5	308:429	5 years
5	Israelsson [13]	1999	50	119	3	26			BMI, Reoperation	59.5	127:95	10 months

Assessment of bias

**Figure 2:**
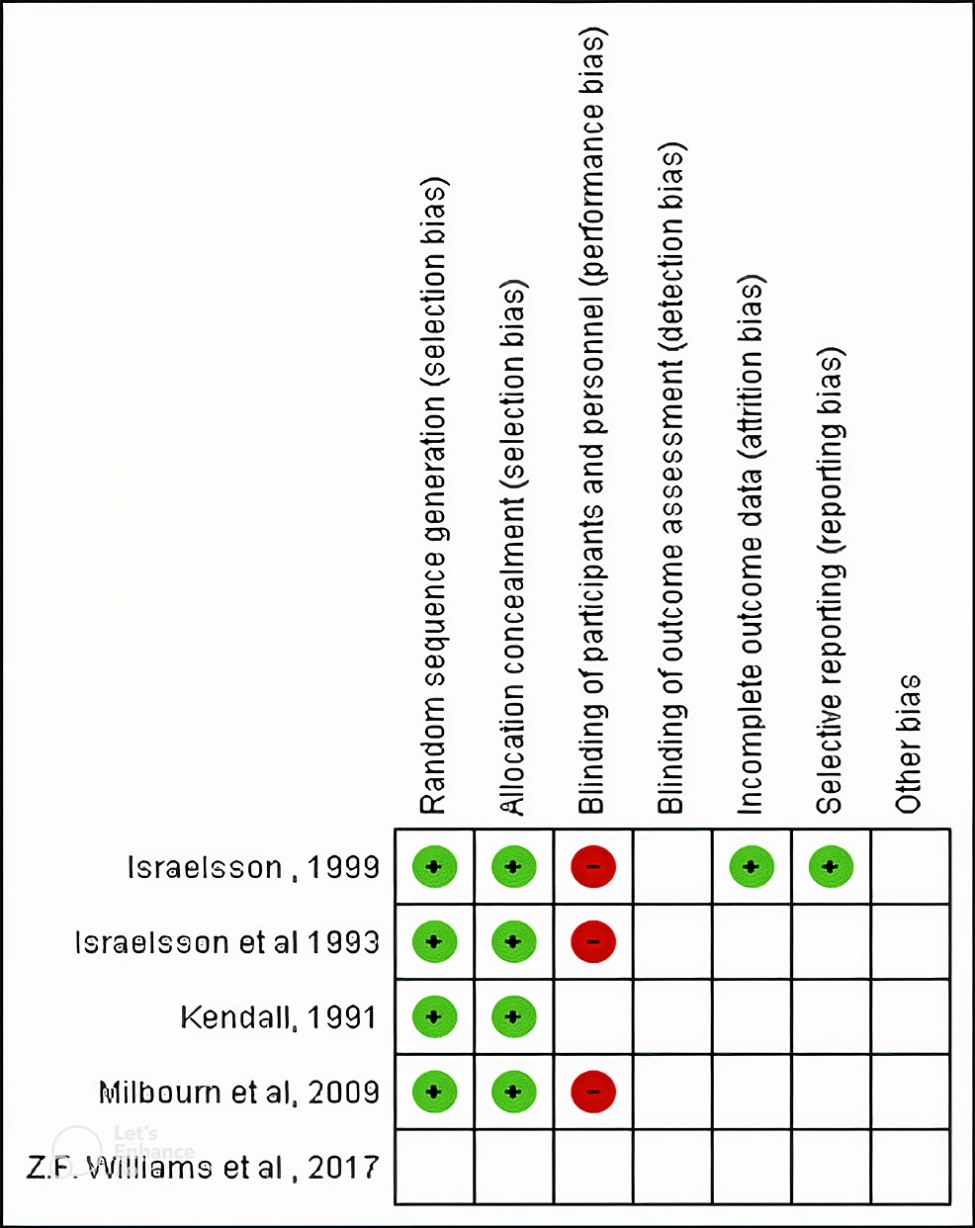
Summary of quality assessment (risk of bias and concerns regarding applicability) for studies included in the meta-analysis according to the RoB 2.0 tool RoB: Risk of bias

**Figure 3:**
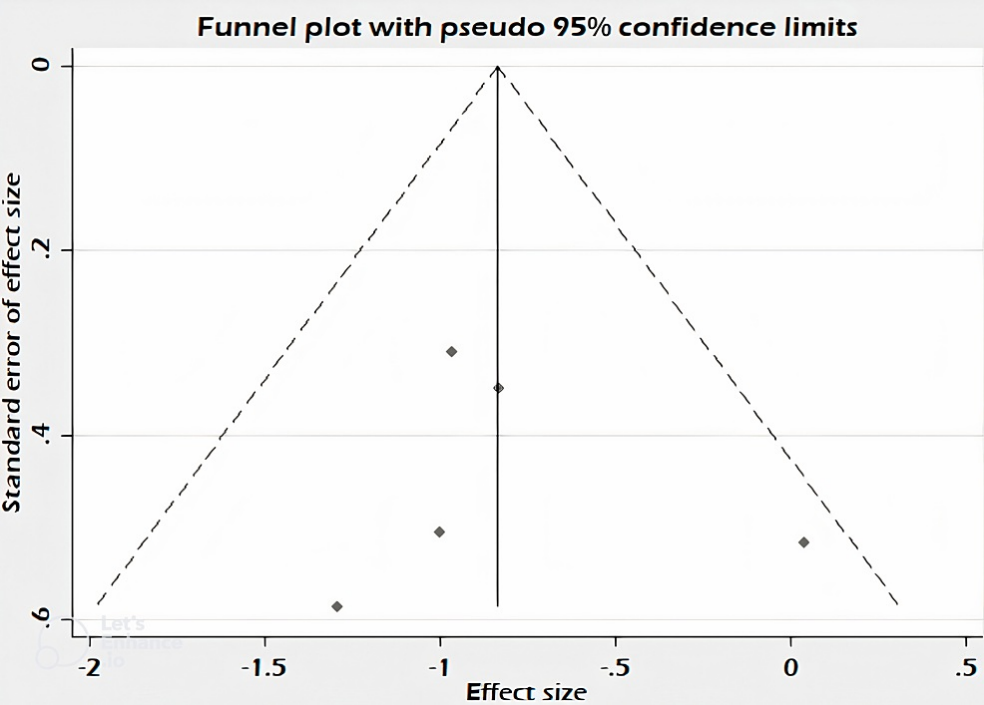
Funnel plot showing publication bias Funnel plot showing asymmetry with p-value 1, 0.779

The assessment of bias was done using the Cochrane risk-of-bias tool for randomized trials (RoB 2.0) tool (Figure 2) in Revman 5.4 software (RevMan, The Cochrane Collaboration, London, UK) [14]. The criteria for assessment were randomization generation, allocation concealment, blinding of the participants and personnel, detection bias, reporting incomplete data, and selective reporting. Due to a limited number of studies selected and analyzed, there was some asymmetry in the funnel plot (Figure 3) [15]. The p-value calculated using Begg’s test was 1 and using Egger’s test was 0.779 which was not significant and showed that there was no publication bias [16].

Primary and secondary outcomes

The primary outcome was the incidence of incisional hernia. The presence of an incisional hernia was ascertained by clinical findings and/or radiological investigations. On clinical examination, any swelling at a previous midline incision scar, which had a positive cough impulse and/or is reducible was considered an incisional hernia [17].

The secondary outcome measured was the incidence of SSI. Surgical site infection was defined as any infection within 30 days at an incision site [18]. Parameters like wound dehiscence, time of operation, length of stay, and stitch interval were not present in every study and therefore could not be considered valid.

Measures of treatment effect

A risk ratio with a 95% confidence interval (CI) for randomized clinical trial was used. Data, like age, which was present in parametric form (mean, standard deviation) was used as is in the meta-analysis. Incidences of incisional hernia and SSI among both groups were expressed as proportion and were analyzed.

Assessment for heterogeneity

Assessment of heterogeneity was done using I-square (I^2^) test based on the Cochrane handbook for systematic review [19]. There were two outcome variables: the incidence of incisional hernia and the incidence of SSI. The heterogeneity of the five studies was assessed. The test for heterogeneity among the studies for the incidence of incisional hernia involved the following: an I^2^ value of 0.00% and a p-value of 0.439 indicated that there is no significant heterogeneity thus showing that most of the studies provide a similar result. All the studies were fairly homogenous. The test for heterogeneity among the studies for the incidence of surgical site infection involved the following: an I^2^ value of 72.6% and a p-value of 0.026 indicated a high degree of heterogeneity in the studies. With an I^2 ^value of >50% and significant heterogeneity, random effect analyses were performed. This assumes that the observed heterogeneity has no correlation with the incidence of SSI.

Meta-regression analysis

**Figure 4:**
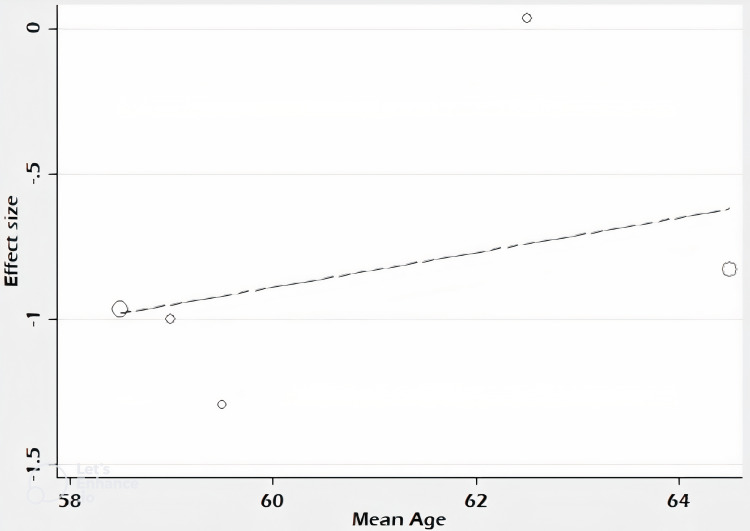
Meta-regression analysis The meta-regression analysis was done with age as a moderator.

It has been seen that the effect size of this meta-analysis increased with advancing age (Figure 4). However, due to the limited number of randomized clinical trials, this was considered not significant.

Statistical analysis

Studies were analyzed using fixed and random effects models. Pooling of data was performed from the randomized controlled trials included. Data were expressed as a mean for the continuous variables, and as a risk ratio for non-continuous variables. The heterogeneity among studies was tested using the chi-square-based Q test. The percentage of interstudy variation was evaluated using the I^2^ value. A p-value of <0.05 and I^2^ of >50% suggested significant heterogeneity among the studies. Publication bias was calculated using Deek’s funnel plot. Statistical analysis was carried out using the STATA software (StataCorp LP, College Station, TX, USA) [20].

Results

There were two outcomes of this review, one was the incidence of incisional hernia and the other was the incidence of SSI. Incidence of incisional hernia was reported in all five studies but SSI was reported in three studies only. The heterogeneity of the results was assessed. Random effect models were used to analyze the results of the incidence of SSI and fixed effect models were used for the incidence of incisional hernia. The following outcomes were measured.

Incidence of Incisional Hernia

**Figure 5:**
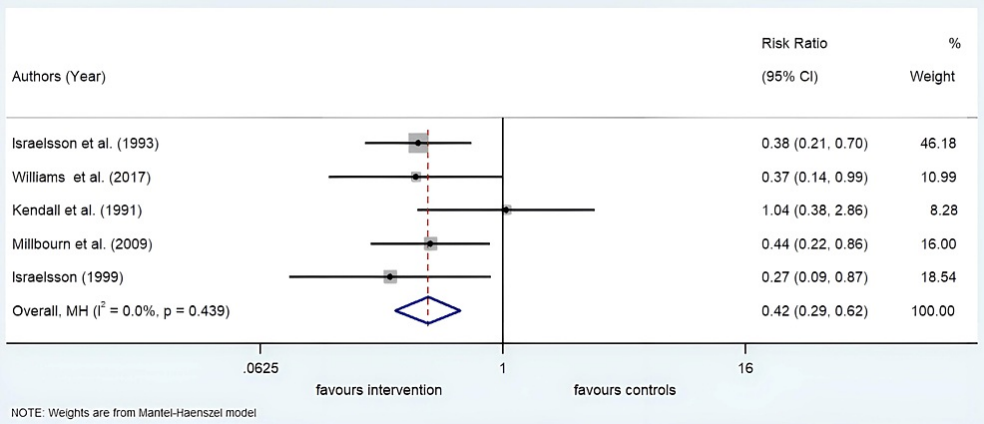
Forrest plot showing the relative risk of developing incisional hernia in the intervention and control group MH: Test of homogeneity

The study population was divided into an intervention group and a control group. The intervention group included patients whose abdominal closure was done with a suture length to wound length ratio ≥4:1. The control group involved patients whose abdominal closuretook place with a suture length to wound length ratio <4:1. The occurrence of incisional hernia was calculated in both the groups in every study. The results of the different studies were quite homogenous. With an I^2^ value of 0.0% and a p-value of 0.439, the degree of heterogeneity between different studies was not statistically significant. Hence, a fixed-effects meta-analysis was performed. The relative risk of the occurrence of incisional hernia if the suture length to wound length ratio was ≥4:1 was 0.42 with a 95% CI of 0.29 to 0.62 (Figure 5). From this, we can deduce that using a short suture length which is less than four times the wound length increases the risk of developing incisional hernia by 2.38 times. 

Incidence of Surgical Site Infection

**Figure 6:**
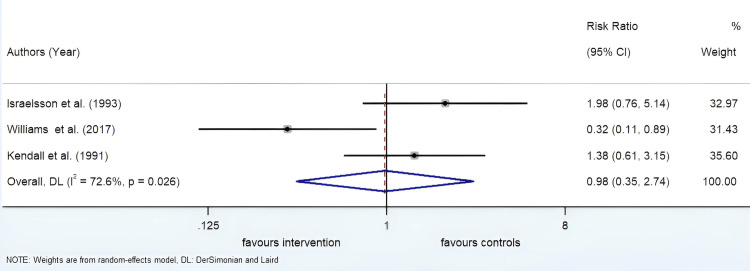
Forrest plot showing the relative risk of surgical site infection in the intervention and control groups DL: DerSimonian and Laird

The secondary outcome was the incidence of SSI. Only three of the five studies had information regarding SSI. With an I^2^ value of 72.6% and a p-value of 0.026, there was a high degree of heterogeneity between the studies. Therefore, a random effects analysis was carried out. The relative risk was 0.98, 95% CI was 0.35 to 2.74 ( Figure 6).

Discussion

This meta-analysis takes into account two postoperative complications of abdominal closure of midline incision after exploratory laparotomy. The incidence of incisional hernia and incidence of SSI following abdominal closure were evaluated. The relative risk of incisional hernia upon closing a midline incision with a suture length to wound length ratio ≥4:1 is 0.42.

The relative risk of the occurrence of SSI in the intervention group was 0.98. With a p-value was 0.966 and a high degree of heterogeneity, the results were not statistically significant. The risk of occurrence of SSI in the intervention and the control group were roughly the same. This shows that using a longer suture length to wound length ratio does not significantly increase the risk of SSI.

Agreements and Disagreements With Other Studies

The findings in this meta-analysis were corroborated by many studies. Beeson et al. acknowledged the importance of using a suture-to-wound length ratio of 4:1 to prevent incisional hernia [21]. He thus wanted to propagate this knowledge by teaching surgery residents the art of proper abdominal closure to prevent incisional hernia. Ceydeli et al. suggested the mass closure of abdominal layers using a monofilament absorbable suture in a running fashion with a suture-to-wound length ratio of 4:1 [22].This particular technique of wound closure prevented early and late complications like wound dehiscence and incisional hernia. Chatterjee et al. in their study found that in patients where abdominal closure was done with a suture length more than four times the length of the wound, using short stitches, had fewer complications [23]. The incidence of SSI and incisional hernias were considerably less in these patients.

In literature searches of various databases and search engines, we could not find a published meta-analysis or systematic review on this topic. Randomized control trials have tried to prove the advantage of using a longer suture length with a short stitch technique without any significant results. Deerenberg et al. conducted the small bites versus large bites for closure of abdominal midline incisions (STITCH)-a double-blind, multicentre, randomized controlled trial-and formulated updated and new guidelines for the safe closure of abdominal wall incisions [24]. They recommended closure using small bites, which implied using a longer suture length to wound length ratio to decrease the tension in wounds and prevent incisional hernias. The incidence of incisional hernia was 21% in the large bite group vs 13% in the small bite group. Effects of the short stitch technique for midline abdominal closure on incisional hernia (ESTOIH), a randomized clinical trial published in 2022, compared fascial closure techniques using short stitch and higher suture length to wound length ratio with using a longer stitch length [25]. The incidence of incisional hernia was more in the long stitch group; however, this was not found to be statistically significant.

Quality of Evidence

This meta-analysis took into account five randomized controlled trials that compared the effects on incisional hernia and SSI by using different suture length to wound length ratios during abdominal closure. These studies were selected upon satisfaction with all the inclusion criteria. Studies from 1991 to 2017 were selected and analyzed. Participants ranged in size from 100 to 363. The follow-up period of the trials ranged from a minimum of 10 months to five years. Four studies were randomized and randomization techniques ranged from drawing a blind card to doing one technique of wound closure on alternate weeks. There was no mention of randomization in one of the studies. Various outcomes were studied, including incisional hernia and SSI in the two groups of interest. When evaluating the incidence of SSI, there was a high degree of inconsistency in the studies. However, upon analyzing the incidence of incisional hernia, the results of the various studies were fairly homogenous.

Bias and Limitations

Due to a scarcity of studies done on this particular topic and the stringent inclusion criteria, only five studies were included. A shorter follow-up period of the studies failed to identify and reflect long-term outcomes. 

## Conclusions

In conclusion, we infer that using a suture length to wound length ratio of more than 4:1 significantly decreases the incidence of incisional hernia. Using a suture length of more than four times that of the wound decreases the risk of developing an incisional hernia by more than half, with a relative risk of 0.42. This method of abdominal closure did not contribute to an increased incidence of surgical site infection. However, an incisional hernia has various other causative factors. Hence, multivariate analysis and well-designed randomized controlled trials may be required in the future to find ways to effectively prevent it.
